# Sustainable development goal 13 and switching priorities: addressing climate change in the context of pandemic recovery efforts

**DOI:** 10.1186/s12302-022-00701-4

**Published:** 2023-01-19

**Authors:** Walter Leal Filho, Aprajita Minhas, Birgit Schmook, Sofia Mardero, Ayyoob Sharifi, Shlomit Paz, Marina Kovaleva, Maria Cristina Albertini, Antonis Skouloudis

**Affiliations:** 1 Department of Natural Sciences, Manchester Metropolitan University, Chester Street, Manchester, M1 5GD UK; 2 European School of Sustainability Science and Research, Hamburg University of Applied Sciences, 21033 Hamburg, Germany; 3 Department for the Observation and Study of the Land, Atmosphere, and Ocean, El Colegio de la Frontera Sur (ECOSUR), Chetumal, 77014 QROO Mexico; 4 School of Geography and Sustainable Development, University of St Andrews, St Andrews, Fife KY16 9AL UK; 5 Graduate School of Humanities and Social Sciences, Network for Education and Research on Peace and Sustainability, and Center for Peaceful and Sustainable Futures (CEPEAS), Hiroshima University, Higashi-Hiroshima, 739-8530 Japan; 6 Department of Geography and Environmental Studies, University of Haifa, 199 Aba Khoushy Ave., Mount Carmel, 3498838 Haifa, Israel; 7 Department of Biomolecular Sciences, University of Urbino Carlo Bo, 61029 Urbino, Italy; 8 Department of Environment, University of the Aegean, University Hill, 81132 Mitilini, Lesvos, Greece

**Keywords:** COVID-19, SDG13, Climate change, Climate finance, Poverty

## Abstract

The COVID-19 pandemic has had many deep social and economic impacts that go beyond health issues. One consequence is that the pandemic has made it even harder to mobilize the financial resources needed to pursue SDG 13 (Climate Action) as a whole and to fund climate change mitigation and adaptation efforts in particular. This is especially acute in respect of the efforts to achieve the targets set by the Paris Agreement and by the recent decisions in Glasgow. This paper looks at how the COVID-19 pandemic has accelerated poverty and undermined climate change mitigation and adaptation efforts, as a result of the switches in priorities and funding. Using a review of the recent literature, an analysis of international trends, and a survey among climate scientists, it identifies some of the impacts of the pandemic on climate change mitigation and adaptation efforts and discusses their implications. The findings indicate a decrease in funding to climate change research since the pandemic crisis. The bibliometric analysis reveals that a greater emphasis has been placed on the relationship between COVID-19 and poverty when compared to the interrelations between COVID-19 and climate change. Addressing climate change is as urgent now as it was before the pandemic crisis started, and efforts need to be made to upkeep the levels of funding needed to support research in this field.

## Introduction: the COVID-19 pandemic and its socio-economic implications

Since the onset of the pandemic, many researchers have worked hard to understand the pathogenetic mechanisms responsible for the development of COVID-19 disease. Understanding the SARS-CoV-2 underlying the infection allows the scientific community to activate therapeutic strategies to counter the pandemic that has been advancing. In fact, to date, the number of infected people and deaths continues to rise [1]. COVID-19 infectious process presents still lacking aspects that prevent the use of effective therapies [2]. For this reason, preventing the entry of the virus, for example with the use of vaccines, seems to be one of the best solutions to keep the pandemic under control. While waiting to achieve vaccination-mediated herd immunity, another effective containment measure is associated with social distancing and lockdowns [3]. Although COVID-19-related restrictions have led to a sudden reduction in greenhouse gas (GHG) emissions and air pollutants, this brief spell will have a modest impact on the crisis according to a new study [4]. However, a post-lockdown economic recovery plan that includes and underlines an environmentally friendly future could make a significant contribution to the fight against global warming. Researchers [4] say the world has a good chance of limiting global warming if governments choose rigid green policies and investments that restore economies following the coronavirus pandemic.

Some researchers reflected upon the challenges of today’s crisis and compiled a list of the similarities and differences between the two crises:

i. High impact trends, with worldwide implications,
ii. Some of the changes caused by them may be irreversible,
iii. Exacerbate social inequalities,
iv. Weakening of international solidarity, and
v. Less costly to prevent than to cure [5].

These similar aspects will have to be taken into consideration to trigger new actions in order to deal with and reduce the impacts of both crises at the same time. In comparison to the COVID-19 crisis which is characterized by immediate severe impacts, the global warming has a slower temporal dimension. Changes in large-scale climate patterns can initiate irreversible processes with unpredictable negative consequences [5]. While the richest countries have the financial ability to invest in reducing the impacts of climate change and rebuilding damaged infrastructures after extreme weather events, the less developed nations are much more vulnerable to the impacts of the climate crisis in terms of water and food insecurity, destroyed infrastructures, increased health problems, among others. In these poor countries, the competition for the limited resources may increase mass migration [5].

The pandemic has affected all sectors [6], and among the primary sector, the agricultural industry has been heavily affected [7]. This was mostly seen through the reduced number of staff available to work, which lowered the levels of trade of some agricultural commodities (e.g., fruits, flowers) in the earlier stages of the pandemic. In other instances, panic buying of food and other basic ingredients led to an increase in demand during the beginning of the first wave; the supply remained limited, causing temporary disarray in the industry [8], which has since stabilized. Furthermore, at the first stage of the pandemic, the primary healthcare services were affected by the shortage of equipment and limited funds to purchase medical resources. Moreover, in developing countries, a lack of medical resources and PPE was observed and is still the case, due to the existing inadequate funding [9].

The manufacturing industry (secondary sector) was also impacted by the pandemic. Levels of international trade were reduced at the beginning of the pandemic, which prevented many products from being manufactured due to a shortage of the necessary materials. The reduction in the levels of manufacturing of goods placed a significant strain on the economies of many countries, thus causing reductions in the gross domestic products of many affected nations [8, 10].

**Fig. 1:**
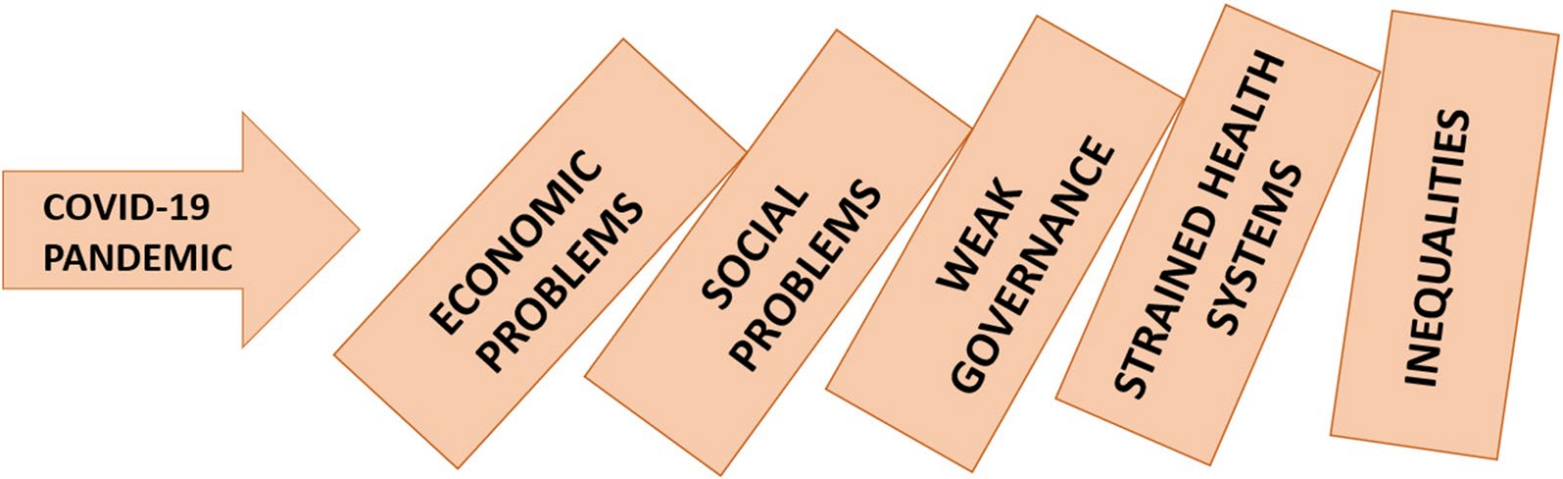
The domino effect of the COVID-19 pandemic

The education sector, at all levels, was among the most affected. The limited ability to have normal classes and the sudden shifting to online platforms has placed a significant strain on students and learners [11]. Other social issues that arose from the pandemic also include increased poverty (belonging to the SDG 1 goal to end poverty everywhere), mental health issues, inequalities towards vulnerable groups, and gender-based violence. The loss of employment of a large percentage of individuals placed many families below the poverty line [14]. Furthermore, people who engage in informal trade saw significant reductions in their income, whereas some received no income at all for a long period, further worsening poverty [15]. Figure 1 presents an overview of the domino effect caused by the pandemic.

A further socio-economic impact is seen in the overall field of health. Apart from the millions of people infected and the clinical cases associated with it, the financial stress triggered by the pandemic caused many people to experience mental health problems such as anxiety or depression [16]. In particular, the loss of family members to the virus and the economic uncertainties caused by the pandemic led to the initiation or worsening of already existing mental health conditions [17]. In other instances, social isolation and staying home for long periods have been associated with increases in violence in households, especially against women [18].

While the COVID-19 pandemic had impacts on finance resources worldwide, this paper looks at how it has accelerated poverty and undermined climate change adaptation efforts, as a result of the switches in priorities and funding. For this reason, the effect of pandemic on SDG 13 research has been analyzed.

## Climate change and the COVID-19 pandemic

Although the rate of the impacts of both the COVID-19 pandemic and the climate crisis is different, both have led to significant health and economic implications [19]. The pandemic has disclosed the fragility of national economies, health care and social systems [20]. In 2020, the global GDP dropped by 3.4% [20] compared to the pre-pandemic forecast of 2.9% growth [21]. The global growth is expected to slow after a fast recovery from 5.5% in 2021 to 4.1% and 3.2% in 2022 and 2023, respectively, amid latest threats from COVID-19 variants and outbreaks, growing inflation, and income inequalities [20]. According to the EU Economic Forecast the EU economy faced a greater reduction of 6.4% in 2020 [22].

**Fig. 2:**
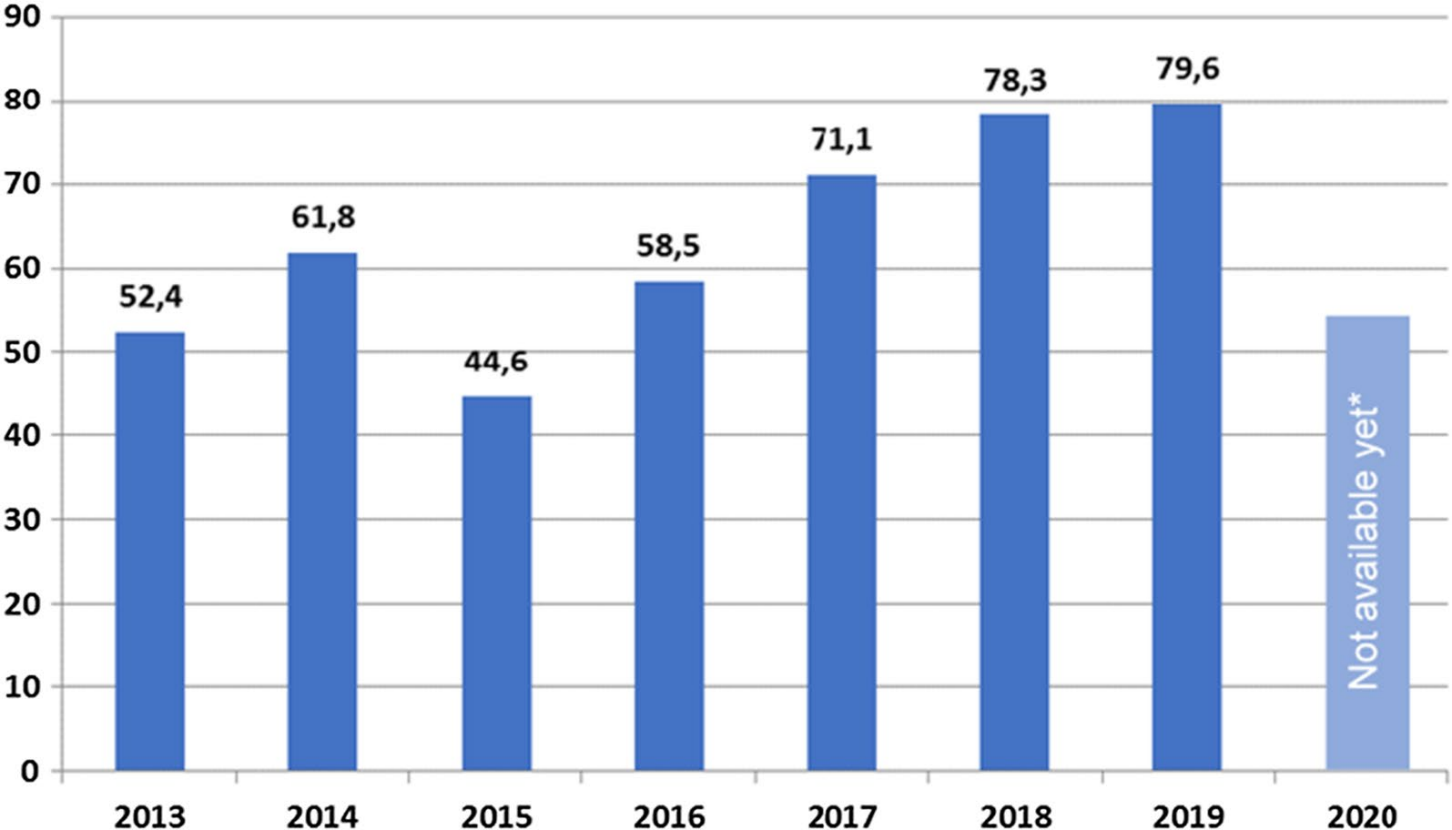
Public and private climate finance provided by the OECD nations between the years 2013 and 2019, USD billion. *Grand totals in 2013–2014 and 2016–2019 are not to be compared directly due to the lack of data on private finance in 2015 and enhanced accounting methods. Data for 2020 are expected to be available in 2022 (Source: OECD [23])

The COVID-19 pandemic, recovery actions, and the post-crisis economic situation might undermine wealthy nations’ priorities and abilities to provide climate finance [23, 24] and alter their green plans, as in the case of China [25]. However, a delay in climate action might even result in a higher cost [26–28]. Even before the pandemic both the private and public sectors were struggling with how to achieve the goal of $100 billion in annual funding to assist low-income nations in combating climate change through adaptation and mitigation measures [23]. Nevertheless, OECD nations’ climate finance contributions have increased in recent years, from USD 58.6 billion in 2016 to USD 78.9 billion in 2018, showing a favorable trend toward achieving the target (Fig. 2. Climate finance provided and mobilized [2013–18, USD billion]). In 2019, the global public and private climate finances that include, among others, flows from financial institutions, governments, companies, and households [29], reached about USD 608–622 billion, which was approximately 15% higher than the amount generated in 2018 [30]. However, this level of financing is not sufficient for the low-carbon transition between 2016 and 2050 and to cover the costs of adaptation between 2020 and 30 [31].

*Furthermore, the national lockdowns are expected to reduce investments in renewable energy projects in 2020 by 10% compared to 2019 *[32], *though this value will vary with the lockdown’s severity *[30]. *Overall, it requires* about USD 2.4 trillion to be invested annually in the energy sector from 2016 to 2035 to stay on a 1.5 °C pathway [33]. Moreover, it was estimated that reaching zero energy-related emissions by 2050 requires an annual global expenditure of USD 1.6 trillion in renewables, electric vehicles, hydrogen, carbon capture, and biofuels technologies [34].

Nevertheless, to support economic activities affected by the COVID-19 pandemic, national governments have introduced a set of stimulus packages that include tax cuts and tax reliefs, benefit payments, grants, and business loans. According to the Greenness of Stimulus Index (GSI), 15 of the announced packages by the G20 will lead to an additional environmental impact or reinforce existing damages [35] by supporting sectors that heavily affect climate change and biodiversity and increase pollution. Currently, the total amount of stimulus has reached USD 12.7 trillion and continues to grow with the introduction of new economic measures [35]. Firms from the carbon-intensive sectors, the majority of which are in Asia, already received USD 866 billion as part of the earlier stimulus programs [30]. Moreover, only 12 of the 300 policies have been implemented, as part of the USD 7.3 trillion stimulus package approved by the G20 nations in April 2020 to reduce long-term CO_2_ emissions [36]. Therefore, COVID-19 and climate change are currently the subject of policy debates, with the goal of reducing economic loss through implementing clean recovery stimulus packages, simultaneously addressing both problems (e.g., [36, 37]). These steps will support the already taken measures and obtained outputs. The ongoing introduction of stimulus measures is seen by experts as an opportunity for national governments to support the decarbonization of their economies by prioritizing such areas as clean energy and physical infrastructure, education and training, natural capital, the energy efficiency of buildings [36] by eliminating subsidies for fossil fuel, and pricing pollution and carbon [38]. Some of the stimulus packages released by major economies do include climate change-related measures that differ in types and dimensions, though the share of the budget assigned for these purposes and implementation frames is not always stated clearly. Examples of some ‘brown’ and ‘green’ measures adopted in stimulus packages of the selected major economies are presented in Table 1 (examples of ‘green’ and ‘brown’ introduced). It should be explained that “brown” measures and processes are those which are based on heavy CO_2_ emissions such as those from fossil fuels, whereas “green” measures are those which are CO_2_ neutral. Currently, the European Commission has introduced the 2021–2027 long-term budget and the temporary recovery instrument, a total of EUR 2.018 trillion (in 2021 prices) which among others includes the most ambitious support of climate-related actions [39]. There are also more efforts to foster economic support to countries, as the pandemic enters its 4th next wave.

**Table 1:** Examples of ‘green’ and ‘brown’ measures introduced in recovery packages of the selected economies

Recovery package	Country	Green measures		References
		Yes	No	
Approx. USD 510 billion relief packages	China	• No strong focus on construction projects that could lead to a spike of CO_2_ emissions • Construction of additional battery-charging and swapping facilities • Promotion of wider use of electric and other clean energy automobiles • Promotion of cleaner and more efficient use of coal • Development of renewable energy • Improvement of systems for the production, supply, and sale of oil, natural gas, and electricity	• Investments in the fossil fuel sectors • Cancellation of civil aviation development fund contributions • Reduction of electricity prices for general industrial and commercial businesses	[25, 40]
Approx. USD 2.16 trillion stimulus package	European Union	• Support of modernization through policies that include research and innovation, among others, via fair climate transition • 30% of the funds for combating climate change • Commitment to propose a carbon border adjustment mechanism • Review of the EU Emissions Trading System		[39]
USD 70.5 billion nationwide economic recovery plan	Russia	One of the main orientations of the plan is sustainable economic growth, but the details have not yet been made public	Tax measures, loans, and government reliefs to support the most affected by the pandemic areas, among which are carbon-intensive industries	[41, 42]
Almost USD 4 trillion, government total support and USD 1.3 trillion as a new package	USA	• Significant investment in solar, wind, and energy storage, grid modernization • Phase-down of the use of hydrofluorocarbons • Weather-proofing of low-income households • Expansion of carbon capture and storage • Improvement of energy efficiency of schools and federal buildings	• USD 58 billion to support the national aviation industry	[43, 44]
1.1 trillion USD a fiscal stimulus package and a new USD 708 billion economic stimulus package	Japan	New package includes spending and initiatives aiming to reduce carbon emissions, including promotion of carbon neutrality, by 2050		[45, 46]
Approx. USD 157 billion stimulus program	Germany	• Promotion of hydrogen technology • Grants to reduce the surcharge levied on electricity consumers Increased investment in the carbon building renovation program • A higher target for expanding offshore wind power • Cancelling of the cap on solar power expansion		[47]
Up to USD 27 billion in direct support to workers and businesses and up to $750 million for a new Emissions Reduction Fund	Canada	New Emissions Reduction Fund to support workers and reduce emissions in Canada’s oil and gas sector, with a focus on methane		[48]

On the other hand, developing countries that already are significantly affected by climate change are facing an unprecedented economic and health crisis. Having limited capacities, the countries struggle to introduce effective recovery schemes and boost their economies. The pandemic not only has deepened inequalities, but also redefined priorities towards addressing climate change.

The objectives of the article are to provide an overview of how the COVID-19 pandemic has accelerated poverty and analyze the extent to which it has undermined climate change adaptation efforts, as a result of the switches in priorities and funding.

## Methodology

The study employed a quantitative research methodology with the help of a survey and bibliometric analysis. To solicit expert opinions on how the COVID-19 pandemic has deepened poverty and undermined climate change adaptation efforts, we conducted an online survey of experts in the field. The online survey included environmental scientists and professionals from the social sciences and humanities working on climate change issues. We included in our online survey not only scientists who work in academia, but also those who work for the government or NGOs, because while these scientists do not always conduct formal research (they often do, however), they do have graduate degrees and often doctorates. Therefore, many NGOs try to access the same funds as scientists working in universities. “Scientist profile” section describes the profile and percentage of respondents from NGOs, government, or academia. The main purpose of the questionnaire was to analyze the effect of the pandemic on SDG 13 research and to find out whether climate financing projects has decreased. For this purpose, we divided the questionnaire into 3 sections: (a) scientists’ background (questions about their country, gender, age, average salary); (b) scientists’ profile (asking about their professional field, the main country where they carry out their research, etc.); and (c) access to climate change research funds (asking if the amount of funding for their research changed since March 2020, among other questions). The link to the questionnaire was spread out by all co-authors to various expert networks in the fields of climate and environmental changes, which, being affiliated with universities and research centers in different parts of the world, such as Germany, the UK, Japan, Mexico, and Israel, allowing for a more global reach. We used “purposive sampling” (also known as judgmental, selective, or subjective sampling, a form of non-probability sampling in which researchers rely on their own judgment when choosing members of the population to participate in their surveys). And we tapped into our networks because we thought this approach made it more likely to get responses. Participation was voluntary and strictly confidential, and this was mentioned to the respondents at the beginning of the survey. The survey was undertaken from 24th February to 7th May 2021.

To gain an overview of the knowledge structure on the interactions between the COVID-19 pandemic, climate change policies, and poverty, we relied on the bibliometric analysis provided by VOS viewer, which is widely used for this purpose [48]. Specifically, we used the term co-occurrence analysis, which provides insight into dominant and common themes and key research focus areas. The input data were obtained from a literature search in the Web of Science. We used the search string provided in Appendix for the literature search. Initially, this returned 407 documents. We examined the abstracts of these documents and selected 358 articles related to the scope of this study for further analysis using VOSviewer. These include 319 research articles, 28 review articles, and 11 letters. The full record and bibliographic details of these articles were downloaded from the Web of Science to be used in the bibliometric analysis. After doing some pre-processing data cleaning to merge synonymous terms (by developing a thesaurus file that was added to the database in VOSviewer), the term co-occurrence analysis was done for a minimum threshold of five terms (i.e., terms that have, cumulatively, been mentioned at least five times in the reviewed articles). The output is presented in the form of nodes and links (see Fig. 5). In Fig. 5, the node size is proportional to the number of times the terms have been used, and link size is proportional to the strength of the connection between terms. Terms that are close to each other in Fig. 5 have co-occurred more frequently and establish thematic clusters that will be further discussed in the results section. Between 103 respondents, many consider themselves either environmental scientists (*N* = 38/37.25%) or individuals engaging in social science and humanities (*N* = 27/26.47%).

## Results and discussion

### Survey results

#### Scientist background

Of the 103 respondents, 63 were males (61%) and 37 were females (36%); 3 individuals preferred not to disclose their sex. Researchers from 49 countries participated in our study, with Germany and the United Kingdom being the countries with the highest participation (8 and 7, respectively). The fact that responses were gathered from scientists from nearly 50 countries provides an overview of the international scope of the problem.

**Fig. 3:**
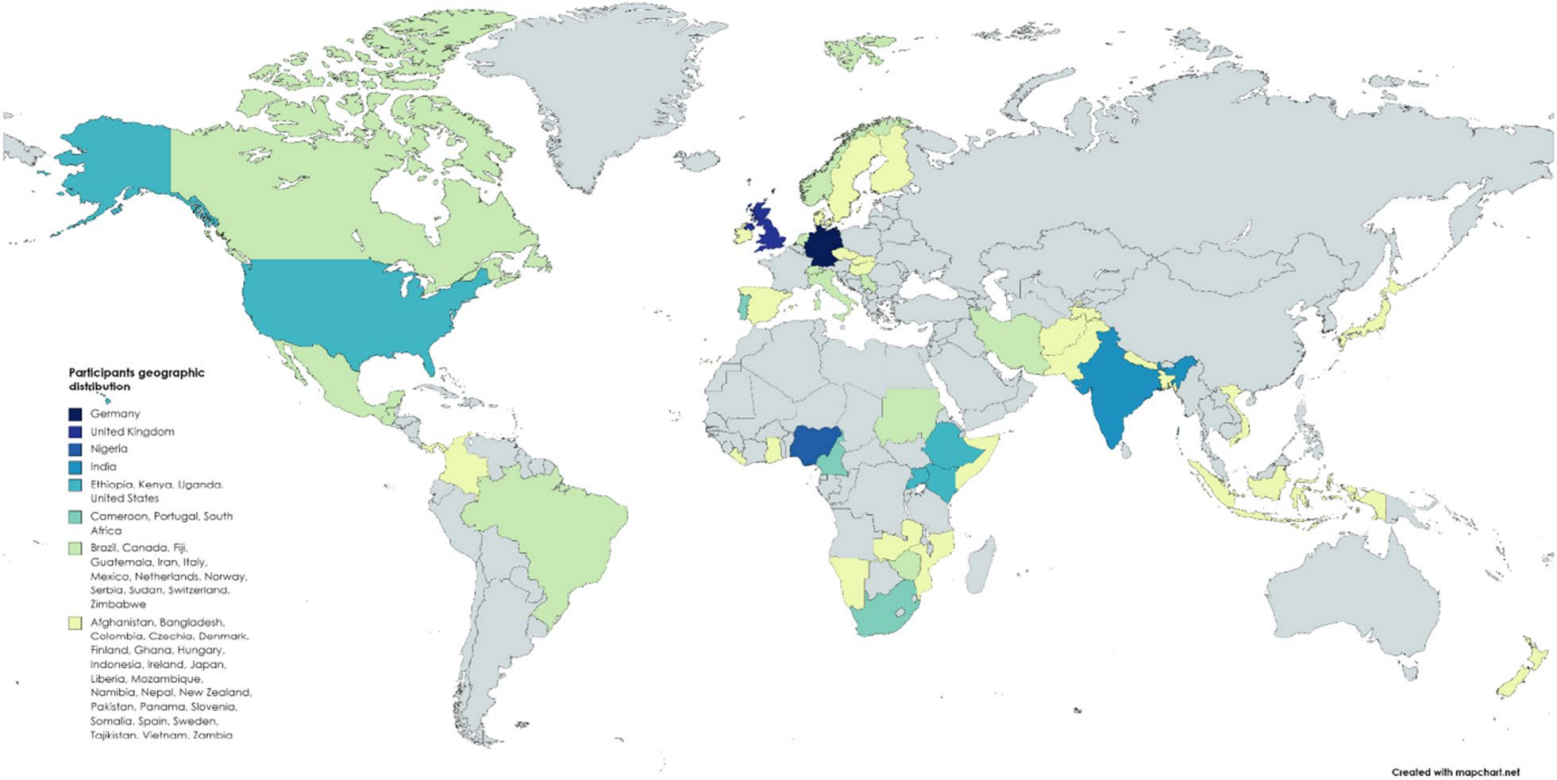
Shows the geographic distribution of the participants

Most of the 103 individuals reside in Europe (*N* = 36/35.29%), followed by individuals from Africa (*N* = 34/33.33%), Asia (*N* = 17/16.67%), Latin America (*N* = 8/7.84%), and North America (*N* = 6/5.88%), with one person (0.98%) from Australia and Oceania as shown in Fig. 3. The respondent’s geographical distribution seems to be a reflection of the authors’ networks, to which the survey link was distributed and considerably underestimates the number of researchers in other world regions, such as North America. The main age group of the respondents was between 41 and 60 years of age, stated by more than half of the participants (51.49%). Most of the 100 responses indicate possession of a post-graduate degree (*N* = 82/82%).

#### Scientist profile

The 103 responses to the scientist profile question indicate that they work for different sectors, with the majority (*N* = 64/64%) being employed in public or private universities, followed by 17 persons (*N* = 17/17%) working in the NGO sector. As for their professional fields, many of the 103 respondents consider themselves environmental scientists (*N* = 38/37.25%), followed by individuals engaging in social science and humanities (*N* = 27/26.47%). Regarding the question about where the research is carried out, a few respondents mentioned that they work in more than one region; therefore, a total of 135 answers were valid. Most of the research is carried out in Africa (*N* = 48/35.56%), especially in the sub-Saharan portion of the continent; the next in frequency is Asia (*N* = 28/20.74%) followed by Europe (*N* = 26/19.26%) and Latin America and the Caribbean (*N* = 24/17.78%).

Regarding the field of expertise in climate change research, several respondents mentioned more than one field (271 answers). Fields of research (see Table 2) span over a wide range, with climate change adaptation and resilience being the most popular, followed by impacts, mitigation, and vulnerability assessment.

**Table 2:** Fields of research of participants

Fields of research	Frequency	Percentages
CC adaptation and resilience	56	20.66
CC and conservation	11	4.06
CC awareness	21	7.75
CC communication and action	23	8.49
CC education	21	7.75
CC governance	2	0.74
CC impacts	42	15.50
CC mitigation	32	11.81
CC projections	5	1.85
CC risk/vulnerability assessment	31	11.44
CC sustainable development and smart livelihoods	27	9.96

#### Access to climate change research funds

Regarding the sources of funding throughout the respondent’s career, of the 98 individuals who provided an answer, 48 (48.98%) mentioned national research councils (public funding) as their main support source for research, followed by International/foreign country councils or corporations (15.31%). Multilateral climate finances are important for another 15 (15.31%) of the respondents, while the remaining 16 (16.33%) listed charitable foundations, self-sponsorship, and undisclosed sources.

When asked whether they had applied for funding after the start of the COVID-19 pandemic in March 2020, of the 103 individuals responding to that question, 66 (64.7%) stated that they did not apply for any funding. Of the 36 respondents who applied for funding, 14 (40%) reported a successful outcome, 11 (31.43%) received a negative result, and 10 (28.57%) do not know yet about the outcome.

The extent to which the amount of funding for their climate change research changed since March 2020 was rated by 103 respondents as follows: 35 (35%) stated that they don’t know; 29 (29%) experienced a subsequent decrease in funding; 29 (29%) experienced no change, and only 7 (7%) found themselves in the lucky situation of increased funding.

Regarding information about whether the researcher’s usual sources of funding stopped or postponed funding for climate change projects since March 2020, of the 96 individuals answering this question, approximately a third don’t know (*N* = 34/35.4%), another third (*N* = 32/33.3%) stated that their usual funding source stopped or postponed funding, while the remaining third (*N* = 30/31.3) said this did not happen. Ten (10.64%) individuals answered that they don’t know if they need to change the focus of their research. As to whether COVID-19 has a direct or indirect relevance to the 99 respondent’s research agenda, the vast majority (*N* = 77/77.8) answered yes, while 22 (22.2%) stated that COVID-19 is not of any relevance for their ongoing research. Of these 22 respondents who stated that COVID-19 has no direct or indirect relevance for their research agenda, we wanted to see which field of research they work in. We obtained 71 responses, as several respondents mentioned more than one field; the three main orientations of their research are adaptation and resilience (16%), risk and vulnerability assessments (14%), and impacts of climate change (13%). These fields of research are also the most popular among the 103 respondents. We also performed an association analysis between these variables (the relevance of COVID-19 in their research agenda with their field of climate change research) and the result showed that there is no statistically significant association between the two (*p*-value = 0.63). The future impact of COVID-19 on their climate change research, as expressed by 100 respondents, is expected to increase according to more than half of the respondents (*N* = 56/56%), to decrease as stated by 13 (13%) respondents, while 15 (15%) expect no change at all, and 16 (16%) don’t know.

#### Associations and dependencies

Taking into account that many of the participants had stated that their funding for climate change projects had changed since the beginning of the COVID-19 pandemic, the next step was to reveal if these changes had any relation with or dependence on other variables, such as the continent where the participants work, their monthly income, their professional field, the orientation of their research, or their funding agencies.

**Fig. 4:**
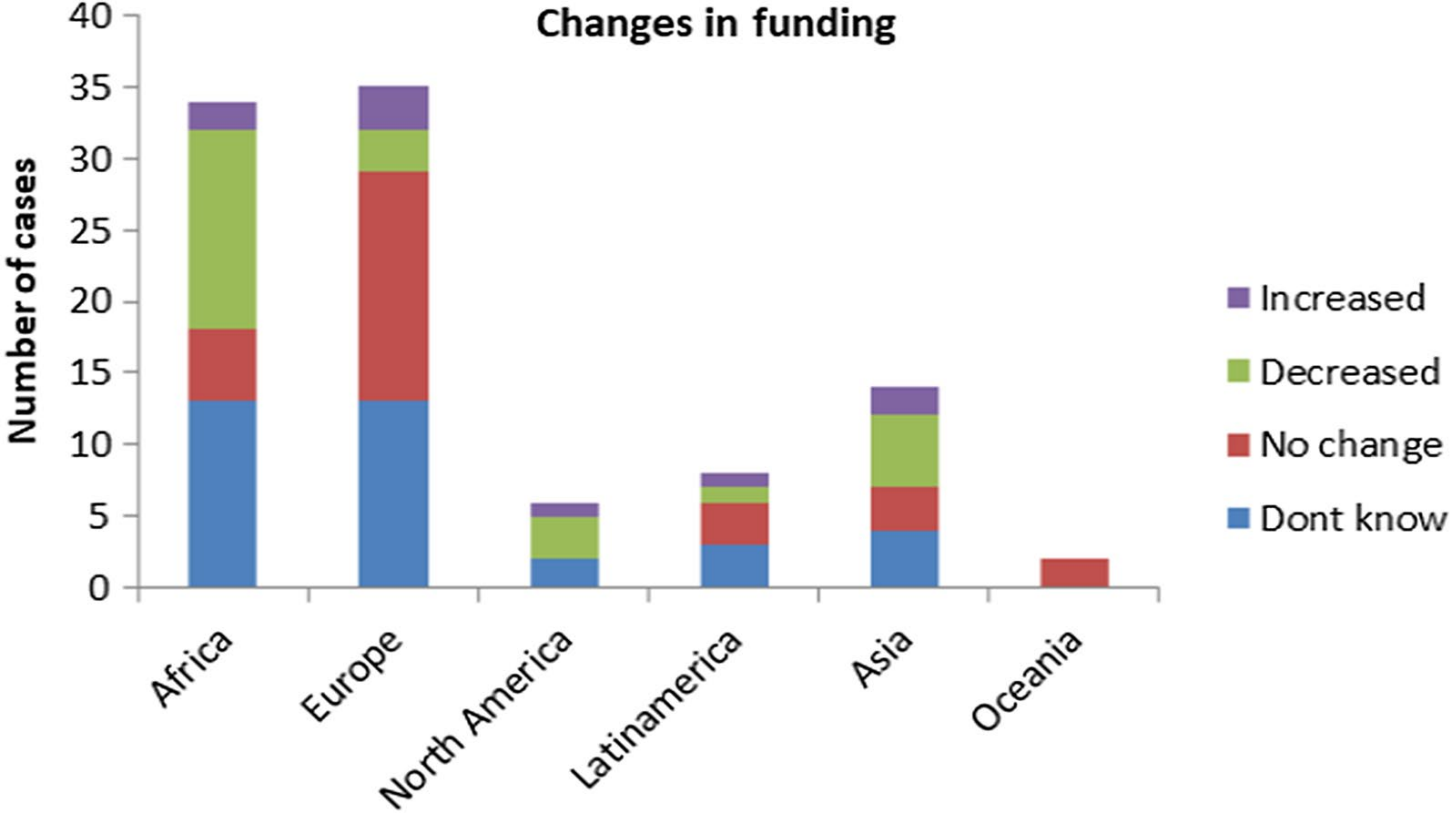
Changes in funding across geographical regions

The following graph (Fig. 4) illustrates an apparent relationship between the participant’s geographic region (continent) and changes in financing. It can be seen that in Africa the majority of the respondents (41%) stated that funding has decreased since the COVID-19 pandemic began.

In Europe, on the other hand, the majority (47%) of the 36 respondents reported no change in funding, 8% reported an increase, only 8% reported a decrease, and 36% did not know.

This may be linked to the Recovery Package of the European Commission, which stated that 30% of the EU funds should be directed towards fighting climate change and fair climate transition, the highest share ever of the European budget.

We could only compare Africa and Europe, as they have a similar *N* (34 and 36, respectively); the differences among the other geographical regions do not allow for such a comparison.

To determine whether there is a statistically significant association between changes in funding and some other variables, we performed a Chi-squares test and a Fisher test applied to four variables separately. The first test, applied to the variables ‘changes in funding’ and ‘researcher’s continent’, showed that there is an association between these two variables, with a *p*-value of 0.024 in the Fisher test. The rest of the variables—funding agency, professional field, and research orientation—did not show an association with changes in funding, since all their *p*-values were above 0.05.

To further explore this significant relationship between the geographical region or continent of the researcher and changes in financing, we decided to perform a MANOVA (multiple analysis of variance), which not only shows us this association but also shows us if there is a dependency between these and other variables.

To calculate the MANOVA, the geographic region (continent) was selected as the independent variable, and the following as dependent variables:

- Monthly income: divided into 7 categories (Low, Low middle, Middle, Upper Middle, Upper Middle, Low high, High, Upper high).
- Changes in financing: two categories (increase and decrease).
- Percentage of change in financing: 8 categories (more than 30% less, between 20 and 30% less, between 10 and 20% less, up to 10% less, up to 10% more, between 10 and 20% more, between 20 and 30% more and more than 30% more).

The results are shown below in Table 3, highlighting the *p*-value of 0.001, which indicates that there is a dependence of the last three variables mentioned with the geographical region of the respondent.

**Table 3:** Results of MANOVA

Lambda	0.507
*F* (observed values)	2.681
GL1	15
GL2	144
*F* (critical value)	1.736
*p*-value*	0.001

*The risk of rejecting the null hypothesis H0 (variables are independent) when true is less than 0.13%

### Interpretation of the results of the bibliometric analysis

The bibliometric analysis shows that connections between COVID-19 and poverty have been, relatively, more discussed than those between COVID-19 and climate change. Accordingly, the results presented in this study could contribute to filling this gap to some extent. The red color in the map is more related to impacts discussed in the context of poverty. It may indicate that the pandemic and associated response measures such as lockdowns have exacerbated poverty and significantly affected livelihood capacities, food security, and mental health of some societal groups. This is in line with arguments in the literature that some groups are disproportionately affected by the pandemic [49]. In terms of connections between COVID-19 and climate change, it is evident that implications for both mitigation and adaptation are discussed in the literature. Most notably, the pandemic resulted in temporary reductions in global GHG emissions due to strict lockdown measures [50]. Also, given its major impacts on the global economy, it has been discussed that the pandemic will have major implications for climate change adaptation and mitigation efforts. This, in particular, has been discussed about economic consequences such as poverty and unemployment. Increased unemployment will further reduce the adaptive capacity of citizens and increase poverty. This may direct the existing governmental budget towards poverty alleviation and reduce the budget needed to implement climate actions, thereby making it difficult to implement mitigation measures such as carbon pricing, which puts more pressure on the urban poor [27]. The stimulus packages designed for economic recovery may also delay actions aimed at achieving climate stabilization targets.

Accordingly, recovery packages should be designed and implemented in a way, whereby economic growth and emissions are decoupled [36]. Furthermore, enhancing adaptation and mitigation prospects requires overcoming the socio-economic inequalities that are further deepened by the pandemic. Indeed, climate change disproportionately affects the poor and vulnerable groups; therefore, reducing inequalities should improve citizen adaptive capacities [27]. Additionally, it can contribute to mitigation, as citizens are believed to better support mitigation policies that they perceive as fair [27]. For instance, fair policies that ensure meeting the needs of poor communities contribute to mitigation by protecting the natural ecosystems and forests (as major carbon sinks) that would otherwise be cut down by poor rural households to meet their livelihood needs. To ensure a green economic recovery that contributes to climate change adaptation and mitigation, it is critical to scale up the investment in clean infrastructure and renewable energy technologies, increase investment in ecosystem protection and restoration, enhance infrastructure efficiency, and take actions towards minimizing COVID-19-induced poverty through capacity building, training, and education programs aimed at reducing unemployment [36].

The reviewed literature has also emphasized that climate change should be addressed with the same urgency as the pandemic, and it has drawn similarities between climate change and the pandemic in the sense that both affect vulnerable and poor groups disproportionately.

Both COVID-19 and climate change are argued to be major health threats that are closely linked and can have intensifying effects on each other [51, 52]. If not designed appropriately, actions aimed at dealing with both pandemics and climate change may exacerbate inequalities. For instance, lockdown measures in the absence of financial support will impact the livelihood capacities of underprivileged groups; similarly, some climate change measures such as carbon pricing affect low-income people disproportionately [49]. Accordingly, government recovery stimulus plans should ensure fair and equitable distribution of resources. This way, the economic distress associated with unemployment will be minimized. As a consequence, individuals are more likely to care about climate issues and perceive them as risks [49].

**Fig. 5:**
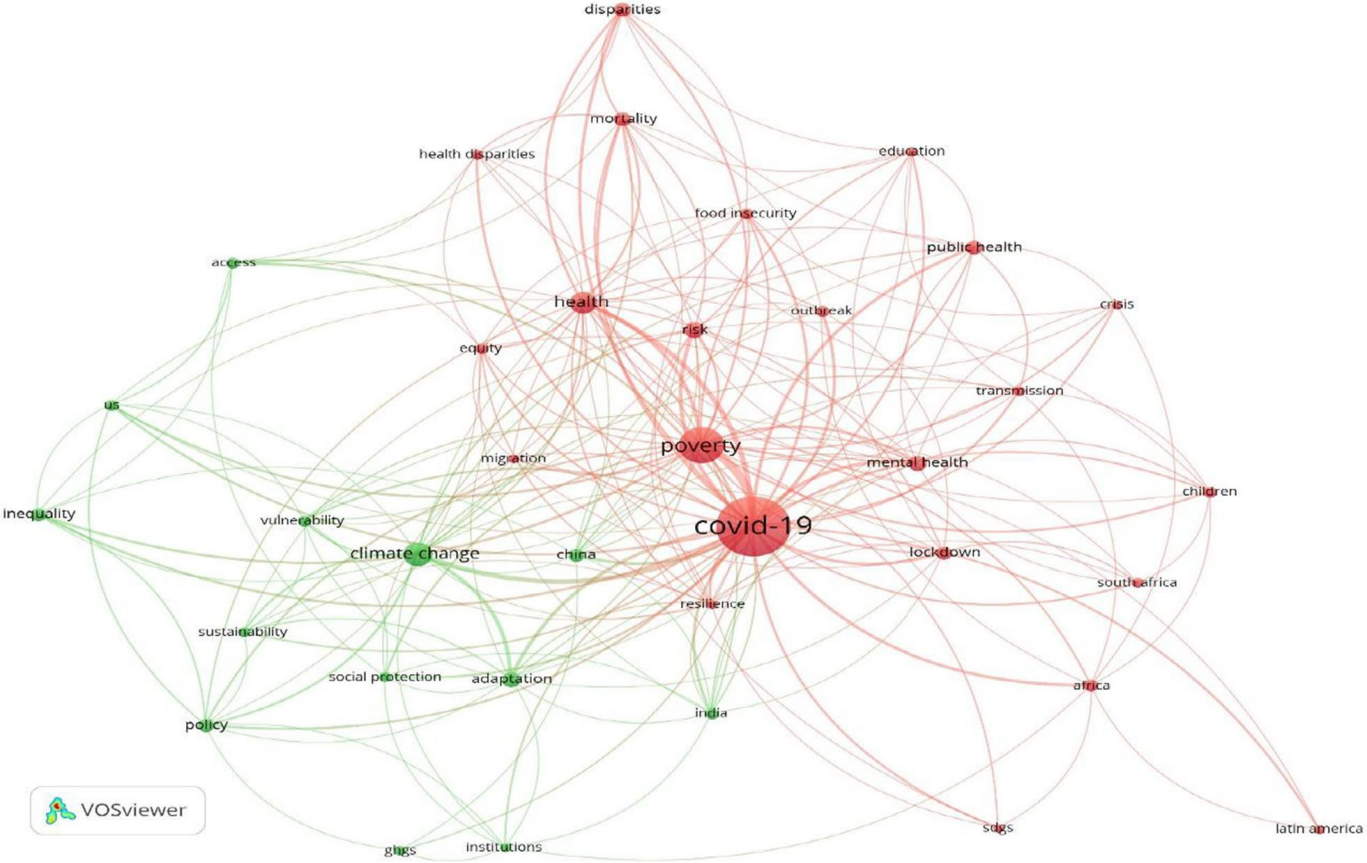
Result of the term co-occurrence analysis

In the term co-occurrence (Fig. 5), the term health also has a central place and is closely linked to other terms such as disparity, equity, poverty, and climate change. This is indicative of the significance of carefully designed health measures, such as universal health coverage for better preparation and response to pandemics as well as climate change impacts. Affordable health coverage ensures universal access to preventive as well as therapeutic healthcare and is essential for dealing with COVID-19 as well as climate-induced risks [53]. Overall, overcoming poverty and reducing inequalities are believed to be key determinants of the success of climate policies as well as pandemic response measures.

## Conclusions

This study employed a mixed-methods approach to shed light on some preliminary findings on the pandemic’s implications for poverty and climate change adaptation efforts, as a result of switching priorities and funding. Utilizing a questionnaire-based survey to gather expert opinion, we attempted to examine whether priorities for climate change initiatives and projects have decreased as a result of the COVID-19 pandemic. The survey results were complemented by a bibliometric analysis to gain an overview of the knowledge structure on the interactions among the COVID-19 pandemic, climate change policies, and poverty.

It is relevant to note that 77.8% of the respondents point out that the COVID-19 pandemic is of particular relevance for their ongoing research, and 56% confirm that the impact of the pandemic crisis on their climate change research agenda will increase in the coming years. Findings also indicate a perceived decrease in funding since the pandemic crisis began for 29% of the respondents, while the funding for 33.3% was stopped or postponed by their respective funding agencies. This is most evident among climate change researchers in Africa, where 41% report a decrease in project funding. Our findings suggest an association between the region, the researchers who are based in and changes in funding, but this warrants further attention and investigation.

The bibliometric analysis reveals that a greater emphasis has been placed on the relationship between COVID-19 and poverty when compared to the interrelations between COVID-19 and climate change. The term co-occurrence analysis suggests that the term ‘health’ has a central place and is closely linked to terms such as ‘disparity’, ‘equity’, ‘poverty’ as well as ‘climate change’. In terms of connections between COVID-19 and climate change, implications for both mitigation and adaptation are examined in the literature. The reviewed literature stresses that addressing climate change is as urgent as the pandemic crisis and that both affect the most vulnerable and poor social groups disproportionately. Likewise, both COVID-19 and climate change are defined as major health threats that are closely interrelated and may have aggravating effects on each other.

The merits of the work reside in the fact that it is one of the few papers which has examined the negative impacts of the COVID-19 pandemic on climate change research, especially in respect of the availability of funding. Also, the data from 49 countries suggest that this is a global trend, as opposed to a regional one.

This paper has some limitations. Firstly, the sample is limited to allow detailed lessons to be taken. Secondly, the promotion of the study was mostly among climate researchers who have access to electronic networks, so many were not aware of the study, despite our effort to spread it better. Certainly, this study places emphasis on the scientific perspective but a wider range of stakeholder groups surveyed in studies such as ours will offer additional, and more comprehensive, insights. The limited duration of the survey also means that the time available for data collection was rather limited.

Despite these constraints, the inputs provided by 103 respondents from 49 countries allow a rough profile to be built, on the impacts of the pandemic on the priorities seen in many nations. This is a welcome addition to the literature since the pandemic is known to have influenced climate change initiatives in a significant way.

**Fig. 6:**
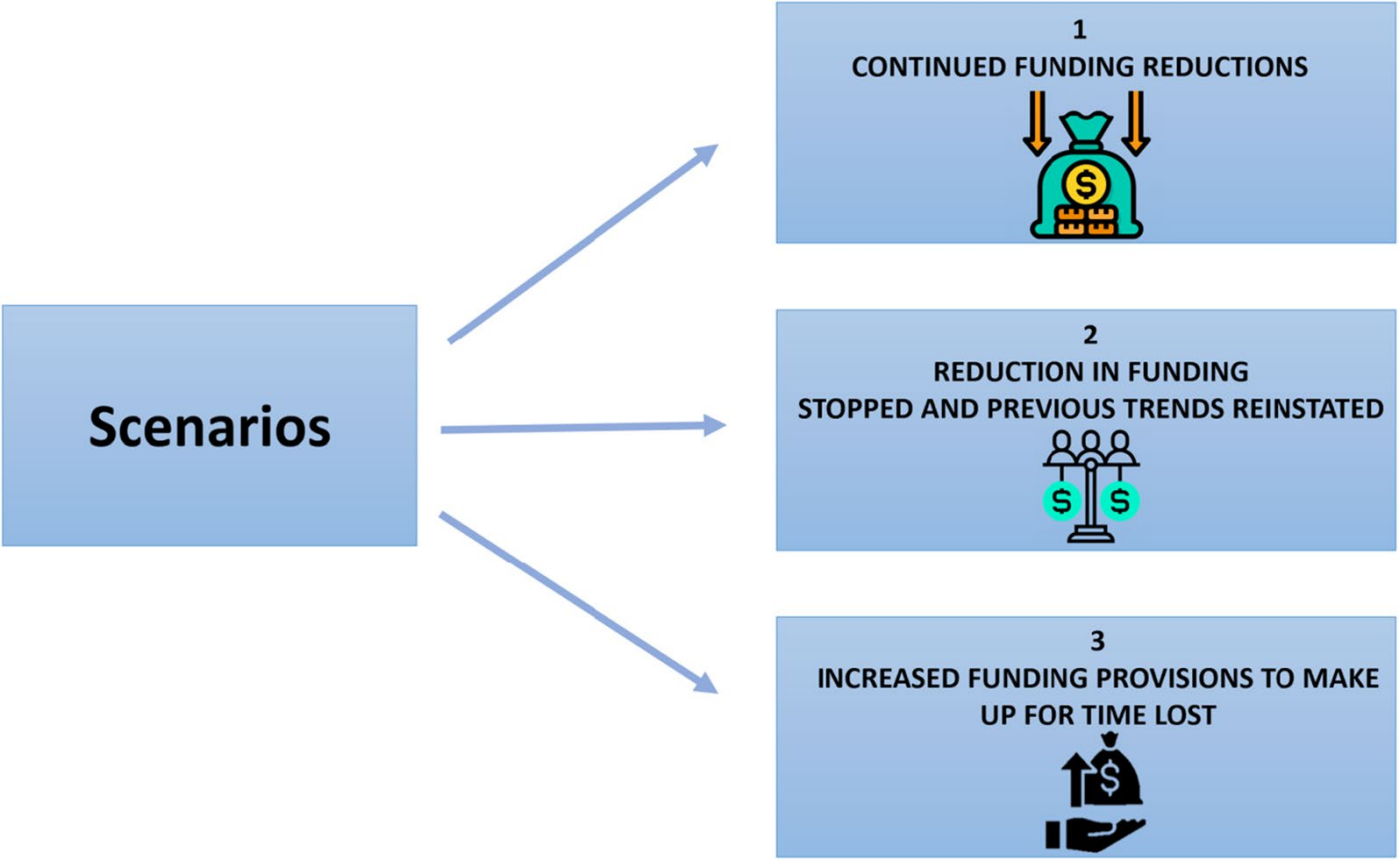
Possible scenarios associated with funding to climate change in connection with the pandemic

The twin crisis of the COVID-19 pandemic, i.e., an economic as well as a health crisis (among many other impacts), has presented mankind with many challenges and has called into question overarching assumptions regarding the achievement of global wellbeing and environmental sustainability. Figure 6 outlines three possible scenarios:

- Scenario 1: Funding to climate change is reduced, further inhibiting efforts to tackle it.
- Scenario 2: Funding cuts are stopped and levels of funding are levelled up.
- Scenario 3: Funding to support climate change initiatives increase.

It is at the moment unclear which scenario is more realistic. Scenario 3 is only realistic if the world economy recovers rather quickly, and the pandemic is fully under control.

In this respect, redefining climate actions under the scope of SDG 13 may provide meaningful insights towards a climate-proof and low-carbon economy in the long term. Whereas the steep drop of global production and consumer demand seen during the earlier stages of the COVID-19 pandemic may have bought us a little time, there is an urgent need for well-aimed responses and to step up efforts in order to prevent the potential of the pandemic to undermine the achievement of the SDGs (especially SDG 13) worldwide. No country or region should be left behind. While the high risk of retreating/rebounding to a business-as-usual normality is evident, policy responses need to drive climate change mitigation and adaptation away from uncoordinated priorities, and towards specific and targets, using adequate indicators. This is particularly important, as the feasibility of achieving the overall SDGs agenda by 2030 has been put into doubt due to the pandemic. Still, the new circumstances around human–planetary systems—especially health—encapsulate new opportunities to build on the work already achieved and recalibrate the drive for sustainability in a post-COVID-19 context.

To this end, further inter- and trans-disciplinary cooperation on climate mitigation and adaptation is needed, to pave the way for the measures which are now being implemented, as the world tries to recover from the pandemic. The importance of climate action cannot be underestimated or ignored, as further inertia may exacerbate the current COVID-19 impacts and could undermine long-term development prospects.

## Data Availability

Not applicable.

## Appendix

See Table 4.

**Table 4:** Shows search terms and items for bibliometric analysis

Focus	Search string	No. of articles
COVID and Poverty	TS=((("covid*") OR ("coronavirus")) AND ("poverty")) *Indexes*=*SCI-EXPANDED, SSCI, A&HCI, ESCI Timespan*=*1900–2021*	358 items

## Abbreviations

COVID-19: Coronavirus disease
CC: Climate change
SARS-CoV-2: Severe acute respiratory syndrome coronavirus 2
GHG: Greenhouse gas
GDP: Gross domestic product
IMF: International Monetary fund
SMEs: Small and medium enterprises
OECD: Organisation for Economic Co-operation and Development
IPCC: Intergovernmental Panel on Climate Change
IEA: International Energy Agency
GSI: Greenness of Stimulus Index
IISD: International Institute for Sustainable Development
SDG: Sustainable development goal
VOS: Visualizing scientific landscapes
MANOVA: Multiple analysis of variance
